# Review of patient-reported outcomes in periprosthetic distal femur fractures after total knee arthroplasty: a plate or intramedullary nail?

**DOI:** 10.1186/s42836-021-00080-w

**Published:** 2021-06-11

**Authors:** Dustin Rinehart, Tyler Youngman, Junho Ahn, Michael Huo

**Affiliations:** Department of Orthopaedic Surgery, University of Texas Southwestern Medical Center, 1801 Inwood Road, Dallas, TX 75390-8883 USA

**Keywords:** Distal femur periprosthetic fracture, Patient-reported outcomes, Open reduction and internal fixation, Intramedullary nail

## Abstract

**Purpose:**

This study reviewed the literature regarding the patient-reported treatment outcomes of using either open reduction and internal fixation (ORIF) with a plate and screw system or intramedullary nail (IMN) fixation for periprosthetic distal femur fractures around a total knee arthroplasty.

**Methods:**

A total of 13 studies published in the last 20 years met the inclusion criteria. The studies included 347 patients who were allocated to ORIF (*n* = 249) and IMN (*n* = 98) groups according to the implants used. The primary outcome measures were the Knee Society Score or the Western Ontario and McMaster Universities osteoarthritis index. The secondary outcome measures included knee range of motion and the rates of complications, including non-union, malunion, infection, revision total knee arthroplasty, and reoperation. Statistical significance was set at *P* < 0.05.

**Results:**

The mean Knee Society Scores of ORIF and IMN groups were 83 and 84, respectively; the mean postoperative range of motion of the knee were 99° and 100°, respectively (*P* < 0.05); the non-union rates were 9.4 and 3.8%, respectively (*P* > 0.05); the malunion rates were 1.8 and 7.5%, respectively (*P* < 0.05); surgical site infection rates were 2 and 1.3%, respectively (*P* > 0.05); the reoperation rates were 9.6 and 5.1%, respectively (*P* > 0.05); and revision rates of total knee arthroplasty were 2 and 1%, respectively (*P* > 0.05).

**Conclusion:**

Based on the patient-reported outcome assessments, both ORIF with a plate and screw system and IMN fixation are well-accepted techniques for periprosthetic distal femur fractures around a TKA, and they produce similar functional outcomes.

## Introduction

The incidences of periprosthetic fractures (PPFs) are on the rise around the globe. The reported incidence of PPFs around a total knee arthroplasty (TKA) stand at somewhere between 0.3 and 2.5% [1–3]. The risk factors include advanced age, diabetes, elevated body mass index, female gender, and anterior femoral notching during the index procedure [4]. The incidence is expected to increase as more TKAs are being performed annually, and the patients continue to live longer with their TKAs [5]. In addition to the cost associated with the management of PPFs, increased morbidity and mortality also pose challenges [6].

The management of PPFs around a TKA demands significant resource input and incurs high healthcare costs [7]. Moreover, most PPFs require surgical intervention [8]. The surgical options include external fixation, open reduction and internal fixation (ORIF) with a plate and screw system, retrograde or antegrade intramedullary nail (IMN) fixation, and the use of a distal femur replacement (DFR) [9]. Currently, there is no consensus regarding the most effective and safest treatment alternatives. The goals of the treatment include early full-weight bearing, independent ambulation, regaining of adequate range of motion of the knee, and minimal morbidities and complications [10].

Currently, there are no physician-directed or patient-focused outcome measurement scale or instruments specifically designed or validated for PPFs around a TKA. In fact, many published reports did not include the functional outcome assessment [11–13]. In TKA, the commonly used assessments include the Knee Society Score (KSS) and the Western Ontario and McMaster Universities (WOMAC) osteoarthritis index. Both scores can be used for assessing the success or failure of TKAs [14, 15].

The purpose of this study was to review the available publications over the past two decades, which included the patient-reported treatment outcomes of distal femoral PPFs around a TKA. The aim of the study was to determine if there were differences between ORIF with a plate and screw system and IMN for periprosthetic fractures around a total knee arthroplasty.

## Materials and methods

**Fig. 1:**
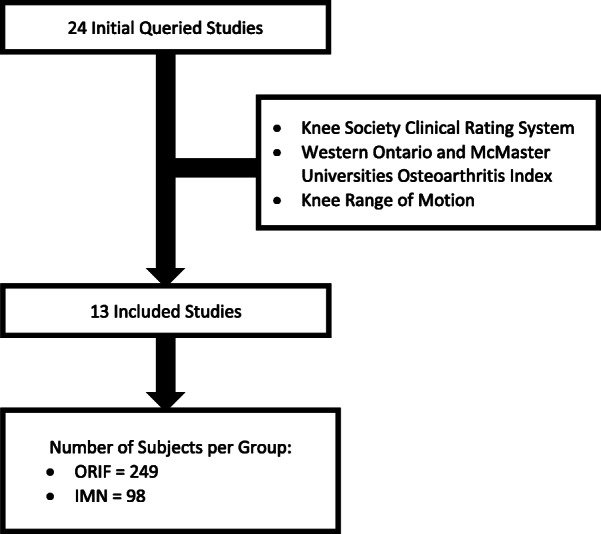
Flowchart diagram of 24 studies

We conducted a literature review by using the key terms “periprosthetic”, “distal femur”, “total knee arthroplasty”, “plate”, and “intramedullary” in PubMed. A total of 24 articles published between January 1, 2000 and January 1, 2020 were retrieved and reviewed for selection. We excluded the systematic reviews and meta-analyses (*n* = 11). We finally included 13 studies for extraction of the relevant primary and secondary outcome measures (Fig. 1). The primary outcome measures were the postoperative patient-reported data consisting of either the Knee Society clinical rating system (KSS) or the Western Ontario and McMaster Universities osteoarthritis index (WOMAC) [16, 17]. The secondary outcome measures included knee range of motion (KROM) and the postoperative complications requiring a reoperation.

The fracture patterns were classified as Rorabeck-Lewis Type 1/2, AO/OTA 33-A1/2/3, Su Type 1/2/3, or Neer Type 2/3 [18]. The type of fracture against each classification system represented a displaced fracture around a stable femoral component of the knee. Because of possible individual variations, the fractures were fixed with either a locked plate and screw system (ORIF group) or an IMN (IMN group).

Descriptive statistics were used to represent the patients' demographics and outcome variables using the average for continuous variables and frequencies. Percentage values were used for the categorical variables. Pooled averages of the continuous variables were calculated by summating the product values of the means and the study populations from each study. Frequency of the complications was presented as percentages cited in each study. *P* values were calculated on the categorical data utilizing the chi-squared and the Fisher tests. A *P* < 0.05 was considered statistically significant. The *P* values could not be calculated on the continuous variables such as KSS scores and the knee ROM given the insufficient data in the literature.

## Results

The population data are summarized in Table 1. The pooled patient variables of ORIF and IMN groups are shown in Table 2. The ORIF group consisted of 11 studies (249 patients) [19–21, 23–26, 28–31]. The mean age was 71.7 years, and 84% of the patients were female. The mean follow-up period lasted 3.6 years. The mean KSS score was 83, the mean WOMAC was 49.5, and the mean knee range of motion was 99°. The IMN group contained 6 studies (98 patients) [20, 22–24, 27, 28]. The mean age was 70.6 years, and 78% of the patients were female. The average follow-up period was 3.6 years. The average KSS score was 84, and the mean WOMAC was 37.1. The mean knee range of motion was 100°.

**Table 1:** Overview of study population of each included study

***Study***	ORIF	IMN	DFR	ORIF ROM°	IMN ROM°	DFR ROM°	ORIF KSS	IMN KSS	DFR KSS	ORIF WOMAC	IMN WOMAC	DFR WOMAC
Agarwal [19]	11	–	–	98.5	–	–	85	–	–	–	–	–
Bezwada [20]	12	18	–	90.0	95.0	–	82	84	–	–	–	–
Gavaskar [21]	20	–	–	106.0	–	–	–	–	–	75.8	–	–
Gliatis [22]	–	10	–	–	–	–	–	–	–	–	58.5	–
Gondalia [23]	24	18	–	96.5	105.7	–	77.2	81.8	–	–	–	–
Kilicoglu [24]	9	7	–	82	82	–	78.8	72.7	–	–	–	–
Kolb [25]	23	–	–	102	–	–	78	–	–	30.2	–	–
Bae [26]	33	–	–	98.9	–	–	84.6	–	–	–	–	–
Lee [27]	–	25	–	–	111	–	–	81.5	–	–	30.2	–
Park [28]	21	20	–	104	100	–	–	–	–	24.4	27.4	–
Kim [29]	32	–	–	103.6	–	–	85.8	–	–	–	–	–
Ha, C [30]	14	–	–	107.3	–	–	78.9	–	–	–	–	–
Darrith [31]	50	–	22	–	–	–	86	–	84	–	–	–

*ORIF* Open-Reduction Internal Fixation, *IMN* Intramedullary Nail, *DFR* Distal Femoral Replacement

**Table 2:** Pooled patient variables by treatment group

***Factor***	ORIF	IMN	DFR
	Value	Value	Value
Age, years, mean	71.7	70.6	74.5
Female, %	84%	78%	86%
Follow-up, years, mean	3.6	3.6	4.0
KSS	83.1	84.2	78.1
WOMAC	49.5	37.1	NA
ROM, °	99.3	100.0	87.0

*ORIF* Open-Reduction Internal Fixation, *IMN* Intramedullary Nail, *DFR* Distal Femoral Replacement, *KSS* Knee Society Score, *WOMAC* Western Ontario and McMaster Universities Osteoarthritis Index, *ROM* Range of Motion

**Fig. 2:**
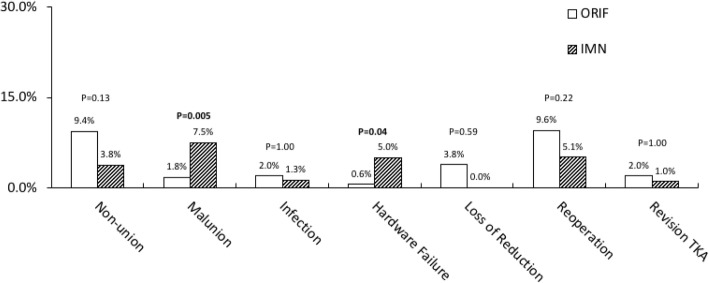
Complications of open reduction and internal fixation (ORIF) and intramedullary nail fixation (IMN) groups

The reported complications in the studies included nonunion, malunion, infection, implant failure, loss of reduction, reoperation, and revision TKA (Fig. 2). The ORIF group had higher rates of nonunion (9.4%), infection (2.0%), loss of reduction (3.8%), and reoperation (9.6%). The complication rates of IMN group were 3.8, 1.3, 0.0, and 5.1%, respectively. There were no significant differences in the rates of nonunion, infection, loss of reduction, and reoperation (Fig. 2). IMN group had a significantly higher malunion rate (7.5% *vs*. 1.8%, *P* = 0.005) and implant failure rate (5.0% *vs*. 0.6%, *P* = 0.04), compared to ORIF group.

## Discussion

For fixing periprosthetic fractures around a total knee arthroplasty, both open reduction internal fixation with a plate and screw system and intramedullary nail fixation produce similar results in terms of Knee Society Scores, Western Ontario and McMaster Universities Osteoarthritis Index, and knee range of motion, as well as the complication rates and functional outcomes of the knee.

The patient-reported outcome assessments are important factors for determining the success and limitations of an intervention. These outcomes are routinely assessed by the patients who have undergone TKAs. The KSS and WOMAC scores are among the most commonly used instruments before and after TKAs. The KSS has previously been validated in total knee arthroplasty patients [15, 32]. It combines both patient-reported responses and surgeon-administered assessments. Higher KSS scores indicate better outcomes [33, 34]. The Western Ontario and McMaster Universities Osteoarthritis index is also validated for assessing pain, stiffness, and the physical function. Higher WOMAC scores are indicative of worse outcomes [35]. Miner et al. [36] demonstrated significantly worse WOMAC scores 1 year after TKA, and the knee movement was less than 95° of flexion, compared to the knees with greater flexion.

The ORIF is a well-established surgical technique for the treatment of distal femur fractures caused by either an acute trauma or PPFs around a TKA, but the clinical outcomes are not uniformly satisfactory [37]. Bezwada et al. [20] compared a cohort of patients treated with ORIF to another cohort treated with IMN fixation. They demonstrated that there was no difference between the two cohorts in the KSS score or knee ROM. They, therefore, recommended the treatment should be determined according to the surgeon’s choice and experience. If the femoral component is of the PS design with a closed intercondylar box, most surgeons will choose ORIF rather than IMN fixation. In addition, if there is a pre-existing total hip femoral stem or other implants in the medullary canal, ORIF with a plate and screw system is more likely selected. Bae et al. [26] conducted a sub-group analysis using either locked plates or non-locked plates to treat PPFs around a TKA. They reported no difference in KSS or knee ROM. The patients treated with non-locked plate had a higher nonunion rate. Our data also showed that the nonunion rate of ORIF was higher than that of IMN fixation, but no statistical significance was found due to a small sample size of patients. The malunion rate of ORIF was lower than that of IMN fixation, because ORIF could achieve a more rigid fixation. The reoperation rates were similar because the small sample size produced a statistical bias.

In TKA, the IMN fixation is partially limited by the femoral component design. In addition, the position of the femoral component may also limit the entry point for a retrograde IMN [38]. Our data demonstrated that the failure rate of IMN fixation was higher. Shin et al. [39] reported that the mismatch between the diameters of the retrograde nail designs and the distal femoral metaphysis of the femur may compromise the stability of fixation, especially in the elderly patients with osteopenic bone quality. The IMN fixation is generally a less invasive procedure, which may be associated with higher KSS scores and knee ROM, compared to ORIF. However, the malunion rate of IMN fixation was higher. Pelfort et al. [40] reported that 23% of patients in their series had more than 10° of extension deformity. A mid-term follow-up showed that the malunion, however, did not adversely affect the clinical outcomes of the patients.

Limitations of this study include the lack of available literature over the past 20 years addressing this topic with inclusion of patient-reported outcome measures. Further study is needed with larger sample size to avoid statistical bias. The study is also limited by the fact that the continuous variable of the pooled analysis could not be statistically compared due to the paucity of the reported data throughout.

## Conclusion

Based on the patient-reported outcome assessments, both ORIF with a plate and screw system and IMN fixation are well-accepted techniques for PPFs around a TKA, and they produce similar functional outcomes.

## Data Availability

All data derived from previously published studies.

## References

[1] Kim KI, Egol KA, Hozack WJ, Parvizi J (2006). Periprosthetic fractures after total knee arthroplasties. Clin Orthop Relat Res.

[2] Parvizi J, Jain N, Schmidt AH (2008). Periprosthetic knee fractures. J Orthop Trauma.

[3] Ricci WM (2015). Periprosthetic femur fractures. J Orthop Trauma.

[4] Canton G, Ratti C, Fattori R, Hoxhaj B, Murena L (2017). Periprosthetic knee fractures. A review of epidemiology, risk factors, diagnosis, management and outcome. Acta Biomed.

[5] Della Rocca GJ, Leung KS, Pape HC (2011). Periprosthetic fractures: epidemiology and future projections. J Orthop Trauma.

[6] Jennison T, Yarlagadda R (2020). A case series of mortality and morbidity in distal femoral periprosthetic fractures. J Orthop.

[7] Reeves RA, Schairer WW, Jevsevar DS (2018). Costs and risk factors for hospital readmission after Periprosthetic knee fractures in the United States. J Arthroplast.

[8] Hoffmann MF, Jones CB, Sietsema DL, Koenig SJ, Tornetta P (2012). Outcome of periprosthetic distal femoral fractures following knee arthroplasty. Injury..

[9] Benkovich V, Klassov Y, Mazilis B, Bloom S (2020). Periprosthetic fractures of the knee: a comprehensive review. Eur J Orthop Surg Traumatol.

[10] Cain PR, Rubash HE, Wissinger HA, McClain EJ (1986). Periprosthetic femoral fractures following total knee arthroplasty. Clin Orthop Relat Res.

[11] Hoellwarth JS, Fourman MS, Crossett L, Goodman M, Siska P, Moloney GB, Tarkin IS (2018). Equivalent mortality and complication rates following periprosthetic distal femur fractures managed with either lateral locked plating or a distal femoral replacement. Injury.

[12] Christ AB, Chawla H, Gausden EB, Villa JC, Wellman DS, Lorich DG, Helfet DL (2018). Radiographic and clinical outcomes of periprosthetic distal femur fractures treated with open reduction internal fixation. J Orthop Trauma.

[13] Matlovich NF, Lanting BA, Vasarhelyi EM, Naudie DD, McCalden RW, Howard JL (2017). Outcomes of surgical management of supracondylar periprosthetic femur fractures. J Arthroplast.

[14] Giesinger JM, Hamilton DF, Jost B, Behrend H, Giesinger K (2015). WOMAC, EQ-5D and knee society score thresholds for treatment success after Total knee arthroplasty. J Arthroplast.

[15] Lingard EA, Katz JN, Wright RJ, Wright EA, Sledge CB (2001). Validity and responsiveness of the knee society clinical rating system in comparison with the SF-36 and WOMAC. J Bone Joint Surg Am.

[16] Insall JN, Dorr LD, Scott RD, Scott WN (1989). Rationale of the knee society clinical rating system. Clin Orthop Relat Res.

[17] Escobar A, Gonzalez M, Quintana JM (2012). Patient acceptable symptom state and OMERACT-OARSI set of responder criteria in joint replacement: Identification of cut-off values. Osteoarthr Cartil.

[18] Su ET, DeWal H, Di Cesare PE (2004). Periprosthetic femoral fractures above total knee replacements. J Am Acad Orthop Surg.

[19] Agarwal S, Sharma RK, Jain JK (2014). Periprosthetic fractures after total knee arthroplasty. J Orthop Surg.

[20] Bezwada HP, Neubauer P, Baker J, Israelite CL, Johanson NA (2004). Periprosthetic supracondylar femur fractures following total knee arthroplasty. J Arthroplast.

[21] Gavaskar AS, Tummala NC, Subramanian M (2013). The outcome and complications of the locked plating management for the periprosthetic distal femur fractures after a total knee arthroplasty. Clin Orthop Surg.

[22] Gliatis J, Megas P, Panagiotopoulos E, Lambiris E (2005). Midterm results of treatment with a retrograde nail for supracondylar periprosthetic fractures of the femur following total knee arthroplasty. J Orthop Trauma.

[23] Gondalia V, Choi DH, Lee SC, Hwang BH, Ahn HS, Ong AC, Park HY, Jung KA (2014). Periprosthetic supracondylar femoral fractures following total knee arthroplasty: clinical comparison and related complications of the femur plate system and retrograde-inserted supracondylar nail. J Orthop Traumatol.

[24] Kilucoglu OI, Akgul T, Saglam Y, Yazicioglu O (2013). Comparison of locked plating and intramedullary nailing for periprosthetic supracondylar femur fractures after knee arthroplasty. Acta Orthop Belg.

[25] Kolb W, Guhlmann H, Windisch C, Marx F, Koller H, Kolb K (2010). Fixation of periprosthetic femur fractures above total knee arthroplasty with the less invasive stabilization system: a midterm follow-up study. J Trauma.

[26] Bae DK, Song SJ, Yoon KH, Kim TY (2014). Periprosthetic supracondylar femoral fractures above total knee arthroplasty: comparison of the locking and non-locking plating methods. Knee Surg Sports Traumatol Arthrosc.

[27] Lee SS, Lim SJ, Moon YW, Seo JG (2014). Outcomes of long retrograde intramedullary nailing for periprosthetic supracondylar femoral fractures following total knee arthroplasty. Arch Orthop Trauma Surg.

[28] Park J, Lee JH (2016). Comparison of retrograde nailing and minimally invasive plating for treatment of periprosthetic supracondylar femur fractures (OTA 33-a) above total knee arthroplasty. Arch Orthop Trauma Surg.

[29] Kim W, Song JH, Kim JJ (2015). Periprosthetic fractures of the distal femur following total knee arthroplasty: even very distal fractures can be successfully treated using internal fixation. Int Orthop.

[30] Ha CW, Shon OJ, Lim SW, Park KH (2014). Minimally invasive plate osteosynthesis for periprosthetic distal femoral fractures after total knee arthroplasty. Knee Surg Relat Res.

[31] Darrith B, Bohl DD, Karadsheh MS, Sporer SM, Berger RA (2020). Levine BR, () Periprosthetic fractures of the distal femur: is open reduction and internal fixation or distal femoral replacement superior?. J Arthroplast.

[32] Dowsey MM, Choong PF (2013). The utility of outcome measures in total knee replacement surgery. Int J Rheumatol.

[33] Noble PC, Scuderi GR, Brekke AC, Sikorskii A, Benjamin JB, Lonner JH, Chadha P, Daylamani DA, Scott WN, Bourne RB (2012). Development of a new knee society scoring system. Clin Orthop Relat Res.

[34] Scuderi GR, Bourne RB, Noble PC, Benjamin JB, Lonner JH, Scott WN (2012). The new knee society knee scoring system. Clin Orthop Relat Res.

[35] Bellamy N, Buchanan WW, Goldsmith CH, Campbell J, Stitt LW (1988). Validation study of WOMAC: a health status instrument for measuring clinically important patient relevant outcomes to antirheumatic drug therapy in patients with osteoarthritis of the hip or knee. J Rheumatol.

[36] Miner AL, Lingard EA, Wright EA, Sledge CB, Katz JN (2003). Knee range of motion after total knee arthroplasty: how important is this as an outcome measure?. J Arthroplast.

[37] Chen F, Mont MA, Bachner RS (1994). Management of ipsilateral supracondylar femur fractures following total knee arthroplasty. J Arthroplast.

[38] Jones MD, Carpenter C, Mitchell SR, Whitehouse M, Mehendale S (2016). Retrograde femoral nailing of periprosthetic fractures around total knee replacements. Injury..

[39] Shin YS, Kim HJ, Lee DH (2017). Similar outcomes of locking compression plating and retrograde intramedullary nailing for periprosthetic supracondylar femoral fractures following total knee arthroplasty: a meta-analysis. Knee Surg Sports Traumatol Arthrosc.

[40] Pelfort X, Torres-Claramunt R, Hinarejos P, Leal J, Gil-Gonzalez S, Puig L (2013). Extension malunion of the femoral component after retrograde nailing: no sequelae at 6 years. J Orthop Trauma.

